# GP Participation and Recruitment of Patients to RCTs: Lessons from Trials of Acupuncture and Exercise for Low Back Pain in Primary Care

**DOI:** 10.1093/ecam/nen044

**Published:** 2010-11-01

**Authors:** Sally E. M. Bell-Syer, Lucy N. Thorpe, Kate Thomas, Hugh MacPherson

**Affiliations:** ^1^Department of Health Sciences, University of York, York, UK; ^2^Medical Care Research Unit, University of Sheffield, Sheffield, UK; ^3^School of Healthcare, University of Leeds, Leeds, UK; ^4^Foundation for Traditional Chinese Medicine, York, UK

## Abstract

The objective of this study was to identify factors associated with general practitioner (GP) participation and the recruitment of people to trials in primary care, based on data from two trials of interventions for treating chronic low back pain. The study was based on data from two randomized controlled trials (RCTs), one involving exercise, the other acupuncture, and subsequent reporting by GPs in a postal questionnaire. The exercise trial achieved 62% recruitment whereas the acupuncture trial achieved 100% recruitment. In both trials GPs most efficient at referring patients were those with a special interest in the subject area, and those known personally to the research team. A follow-up GP questionnaire found that both trials had maintained a high profile with over 80% of GPs, and successful recruitment strategies included project reminder letters, updates and personal contacts. Achieving target recruitment of patients in the acupuncture trial was aided by the deliberate application of lessons learned in the exercise trial, in particular the need to keep initial study entry criteria broad, with subsequent filtering undertaken by the study researcher. In addition the use of effective methods of maintaining the trial profile, the involvement of a GP advisor, the decision to maximize the recruitment of GPs early in the trial and the direct recruitment of interested individual GPs. The successful recruitment of patients to trials in primary care requires careful planning and continuous monitoring from the outset. Prior to starting recruitment, it is useful to identify previous trials in a similar environment in order to learn from their experience and optimize patient recruitment.

## 1. Background

Well-conducted randomized controlled trials (RCTs) provide good evidence for the effectiveness of different methods of managing specific conditions. This evidence is an invaluable tool for general practitioner (GPs) in primary care when making clinical decisions with respect to appropriate management. Therefore, a key aspect to successful completion of a trial is the recruitment of patients and there are important issues around planning a trial and its subsequent conduct. However, GPs are not always willing to participate in research studies and when they do agree to participate, many are not effective and successful recruiters [1, 2].

A number of factors that reduce the willingness of GPs to participate in trials and to refer patients have been cited in the published literature. If the research question is not of sufficient interest or not relevant to general practice then GPs are unlikely to take part [3], as they receive many invitations to become involved in research projects. The process of obtaining informed consent can also be an important barrier to GP participation [4, 5], as is the worry that taking part in research could disrupt the “normal" doctor-patient relationship [6]. Lack of financial incentives may also deter GP involvement [3].

Probably the largest barrier to GP participation is lack of time, the daily demands of a busy practice leave GPs with little time to make recruitment a priority [7–10]. Complex protocols and data collection forms and poorly defined study entry requirements are additional factors that discourage GP involvement and can limit patient referrals [8, 11]. In a recent survey of GP participation in trials, forgetfulness was cited as one of the main reasons for not referring eligible patients to the research centre [5].

However, successful recruitment can be encouraged by employing a number of strategies. GP participation in trials can be encouraged by addressing a relevant research question, the outcome of which is applicable to general practice [9]. GPs are more likely to participate in trials if their practice lacks the service that the trial intervention provides and are interested in investigating the effect this intervention will have on managing the patients' condition [7]. Involving GPs in the development of the trial protocol [4, 8] and employing a GP advisor as part of the trial team to facilitate access and to brief potential GP participants can also enhance recruitment [12].

It is recommended that as many GPs as possible agree to participate at the start of trial recruitment in order to establish a sense of ownership to the project [13]. Several authors [7, 8, 13] have discussed traditional methods of approaching GPs, by letter and telephone contact. The involvement of key staff such as practice managers is also encouraged to promote and maintain the trial profile [2, 13].

GPs who have local contacts with the research team are more likely to participate in a trial [7]. Similarly GPs who are particularly interested in either the specific research topic [14], or have a commitment to research in general [9], may be easier to recruit and it is worthwhile targeting these GPs as involved and enthusiastic GPs can then attempt to persuade their colleagues to participate in the research [10]. Financial incentive is another approach that may be useful in increasing GP involvement in research [4, 14].

Several authors have stressed that in order to encourage GP participation in trials and to maximize patient referrals, the demands on both GPs and patients should be kept to a minimum by providing clear guidelines and simple data collection forms [3, 9, 15]. Bell-Syer and Klaber Moffett [13] found that asking GPs to refer patients according to broad study entry criteria and then allowing researchers to establish eligibility and obtain informed consent, was a useful tactic and encouraged patient referrals.

Having succeeded in involving GPs for the trial, it is imperative that researchers maximize referrals by maintaining regular contact with GPs throughout trial recruitment. Personal visits and telephone calls throughout the recruitment period, in addition to regular study updates and project reminders all serve as useful prompts in encouraging patient referrals [7–9, 13].

As an alternative to making individual referrals at the time of the consultation, researchers may consider retrospective recruitment which involves contacting patients directly. These patients are identified through GP database searches and approached by the research team having obtained the necessary permission of the GP [13, 16].

There have been three recent systematic reviews of strategies designed to improve recruitment to trials. One, Watson and Torgerson [17] was concerned only with trials of strategies in secondary care, the second Mapstone [18] focuses on strategies aimed at patients and makes the point that they were disappointed not to find any trials of interventions aimed at clinicians. They state that “motivations and fears of taking part are different” for clinicians. The third, Rendell et al. [1] identifies studies in primary care settings, none of which were RCTs. They found two studies with negative association between personal acquaintance with the researchers as a reason for participating in the trial and subsequent recruitment to the trial. They suggest this might be because clinicians felt obliged to say “yes” to the initial request. They conclude that they are unable to draw clear conclusions from the studies due to heterogeneity of design and intervention, and strongly recommend the use of pilot studies to test strategies in particular contexts prior to commencing full recruitment.

Researchers invariably benefit from the experiences of earlier trials which have been conducted in a similar environment and it is useful to maximize the success of various strategies whilst guarding against spending time and money on plans which are unlikely to achieve an increase in recruitment. The aims of this study were as follows:

1. to compare the experience of patient recruitment by GP in two RCTs conducted in primary care for treating low back pain, and
2. to identify the key points for enhancing recruitment through GP referral.

## 2. Methods

Two RCTs of the management of low back pain, one evaluating exercise [19] and the other acupuncture [20], compared to usual GP management, were conducted in primary care in the York area. A summary of the methods of the two trials, including recruitment, are shown in Table 1. Practice characteristics associated with the GP practices who recruited the most patients to the trials are shown in Table 2. Information was obtained directly from the trial coordinators and data retrieved from original trial reports therefore ensuring accuracy.

### 2.1. GP Postal Questionnaire

In both trials, a postal survey of all participating GPs was conducted after patient recruitment to the study had ceased. The survey questionnaire was designed by the coordinator of the Exercise trial and adapted slightly for the Acupuncture trial and took the form of a short questionnaire asking GPs to report the following:

1. Factors that might have encouraged patient referral to the trials.
2. Factors that might have prevented patient referral to the trials.
3. Their feedback on the main entry requirements for referring patients to the trials.
4. The effectiveness of the methods used to maintain the trial profile in order to encourage recruitment.

The data were analysed on a descriptive basis only.

## 3. Results

### 3.1. GP and Patient Recruitment

The Exercise trial involved 87 GPs from 19 practices in York, and had a recruitment target of 300 patients. Over a 2-year recruitment period 187 patients were randomized to the trial and this represented 62% of the target. One practice referred patients through a computerized listing method, and this practice contributed 44% of the included patients. The Acupuncture trial involved 39 GPs from 18 practices in York, and successfully randomized its target of 240 patients (100%) within the planned 18-month recruitment period.

### 3.2. Patient Referral by GP Practice

Table 2 presents data on the eight GP practices that referred the most patients into the two trials. Interestingly, for the Exercise trial, the top four referring practices (contributing 64% of all patients) were either: practices involving GPs with a special interest in back pain; or practices where the GPs were personally known to members of the research team. One of the practices that involved GPs with a special interest in back pain was also the practice which contributed 44% of the study patients through the computerized listing approach. Similarly the five top referring practices for the Acupuncture trial were either: practices involving GPs with a special interest in back pain; or practices where the GPs were personally known or a GP advisor to members of the research team; or a practice involving GPs who were members of the British Medical Acupuncture Society (BMAS). These five practices contributed 71% of the patients to the trial.

### 3.3. GP Postal Questionnaire

The survey of GPs involved in the Exercise trial elicited 64 responses from 87 GPs while the survey of the GPs in the Acupuncture trial received 27 responses from 37 GPs, an identical response rate of 73% in both trials. The key data relating to factors that impacted on GPs willingness to refer patients into these trials is presented in Table 3.

#### 3.3.1. Main Factors which Reduced GP Willingness to Participate by Referring Patients into the Trials

The main factors which reduced GPs willingness to refer patients, in order of decreasing priority were: that patients were already receiving other ongoing treatment modalities, that not all patients would receive the intervention because of randomization, personal time constraints within the consultation, and the difficulty for patients in travelling to the exercise class/acupuncture clinic.

#### 3.3.2. Main Factors which Increased GP Willingness to Participate by Referring Patients into the Trials

Factors which encouraged GPs to refer patients into the two trials were: the belief in the benefits of exercise/acupuncture for back pain, a desire to support research, the fact that the intervention provided an additional treatment option and positive feedback from existing study patients about the intervention.

#### 3.3.3. Feedback on the Main Entry Requirements for Referring Patients into the Trials

In the Exercise trial, 43% (25/58) of GPs had difficulty remembering the study entry criteria for patients, and 25% (14/57) had difficulty applying these entry requirements. As a result, the Acupuncture trial was advised by the Exercise trial to keep the study entry requirements for GPs as broad as possible and to use the trial researcher to apply the more detailed criteria before entering the patients into the trial. As a result only 19% (5/27) of GPs in the Acupuncture trial had difficulty remembering the study entry criteria and only 4% (1/26) had difficulty applying these to patients.

#### 3.3.4. Effectiveness of Maintaining the Trials Profile in Order to Support Recruitment

The survey revealed that a high profile had been maintained with over 80% of GPs in both studies. The following recruitment strategies were reported as particularly successful in both trials: project updates, project reminder letters, and maintaining regular contact with GPs either by personal practice visits or by telephone. Patient acknowledgement and discharge letters were also reported as useful in the Acupuncture trial.

#### 3.3.5. Financial Incentive

There is no evidence to suggest that the financial incentive offered by the Acupuncture trial (*£*5 per patient) aided GP involvement and patient referrals. It was not possible to test the impact of incentives directly; however in the GP survey not one GP reported this as being a factor that encouraged them to refer patients into the trial. It was recognized this incentive was of a low monetary value and its use in the Accupuncture trial has not contributed to the evidence base.

## 4. Discussion

From the literature and from the experience of two similar trials we have identified factors that are associated with GPs willingness and/or reluctance to participate in, and to refer patients into clinical trials. From the literature, it is clear that GPs are more likely to participate if there is a relevant and interesting research question. Both clinical trials investigated the management of a condition of particular relevance to general practice-low back pain. According to the findings of a recent OPCS survey quoted in the report of the Clinical Standards Advisory Group (CSAG) on Back Pain [21], the annual cost of back pain to the NHS is estimated at approximately *£*480 million with the annual cost to a GP practice with a list of 10 000 patients estimated at *£*88K [15]. As well as addressing a relevant research question, both trials were evaluating interventions that were not routinely available from the local general practice. This encouraged GP involvement, and is supported by the findings of the GP survey. In both cases the process of recruiting GPs through a letter of introduction, followed up by a practice visit to encourage GPs to refer patients, possibly because it made GPs feel committed to the study and enabled any problems or concerns to be dealt with in the early stages.

In line with the experience from the literature, and in recognition of GPs busy schedules, demands on GPs were kept to a minimum in both trials. Patient referral forms were short and asked for essential information only to be recorded and informed consent was obtained by the researcher rather than the GP. The computerized listing approach of obtaining referrals adopted by the Exercise trial enabled patients to be identified outside of the consultation and reduced the personal involvement of the GP. This retrospective method of recruiting patients is becoming more popular as computerized record keeping increases. However, it will not always be possible or be the recruitment method of choice.

Both trials succeeded in maintaining personal contact with GPs throughout the recruitment period, in order to combat forgetfulness on the GPs part and to maximize patient referrals. Project updates, project reminder letters, personal practice visits and telephone calls were the most successful strategies according to the findings from the GP survey.

There are a number of possible explanations as to why the Acupuncture trial achieved its target number of patients on schedule. First, the Acupuncture trial had the involvement of a GP advisor to aid GP recruitment, something the Exercise trial did not have. The GP advisor encouraged approximately 22% of GPs (28/126) in York to become involved with the Acupuncture trial and refer patients. The practice where the GP advisor was a Principal was also one of the top recruiters for the trial.

Secondly, the Acupuncture trial was advised by the Exercise trial to recruit as many GPs as possible early on in the study, and not to rely initially on a few key GP practices with further practices being recruited later. In the Exercise trial, those GPs recruited later in the trial tended to refer fewer patients, possibly because they felt less involved with the study and had less sense of ownership. The Acupuncture trial followed this advice and recruited their GPs in two waves at the beginning of the trial. As a result, the GPs were more efficient at referring patients throughout the 18-month recruitment period. Findings from both trials revealed that GPs with a special interest in the subject area or GPs known personally to the research team were significantly better patient referrers. It is therefore important to target these GPs as early on in the study as possible. Another difference between the trials is that the Exercise trial recorded recruitment by GP practices, whereas the Acupuncture trial recorded the recruitment by individual GPs, although in reality it was the same individual GPs within practices that made regular referrals.

Thirdly, the Acupuncture trial was advised by the Exercise trial to keep the criteria to be used by GPs to identify potential patients as broad as possible, with subsequent screening by the researcher, and this aided patient referrals into this study. This is supported by the experience of the Exercise trial in which those practices that referred patients paying attention only to the broader entry criteria, and using the researcher to establish eligibility, were much more efficient at recruiting eligible patients than those practices who followed the more detailed entry criteria.

The results of this study must be interpreted in the context of recruitment of low back pain patients in primary care. It should be noted that other factors, not covered by this study, may have impacted on recruitment rates. For example, patients will have perceived the offer by GPs of participating in a trial of prescribed exercise differently than a trial of acupuncture. Prescribed exercise might have been seen by patients as something that they could access on their own relatively cheaply, and this may have reduced the recruitment rate for this intervention. In addition, our data is based relatively small numbers of GPs being surveyed and the response rate may have introduced some response bias. Responses may be subject to a social acceptability bias, for example, citing “forgetfulness” as the reason for not introducing patients to the study, in addition, opinions were sought from GPs via surveys undertaken after recruitment had been completed and therefore there may be some risk of some post-hoc generalizations about what was important to them. Whilst considering these limitations we achieved a sufficiently high response rate in both surveys (73%) for us to be satisfied that we did capture a reasonable picture of the experiences and opinions of the participating GPs.

## 5. Conclusions

Evidence for recruiting patients to trials in primary care is still mixed and often contradictory, also issues may be different dependant on whether the setting is in primary or secondary care.

More research is needed looking at particular strategies to improve clinician participation with adequately powered RCTs. However, given the heterogeneity of study designs, setting and conditions, the recommendation of piloting methods of clinician recruitment and retention might be the best approach [1]. Our study transferred lessons learned from one trial involving a similar patient group and the same pool of GPs and this can be seen as a kind of pilot. As intended, it was associated with a highly successful recruitment rate in the second trial.

## Figures and Tables

**Table 1:** Comparing the methods, including recruitment, in the exercise and acupuncture trials.

Profile	Exercise trial	Acupuncture trial
Brief summary of trial	RCT of progressive exercise programme compared with usual primary care management for patients with low back pain.	Pragmatic RCT evaluating the clinical and economic benefits of offering acupuncture to patients with low back pain, compared to usual GP management.
Inclusion criteria	Age 18–60 years; mechanical low back pain of at least 4 weeks duration but less than 6 months, declared medically fit by their GP to undertake the exercise.	Age 20–65 years; presenting with low back pain or sciatica; up to 12 months pain in current episode and greater than 4 weeks duration Assessed as suitable for management in primary care;
Exclusion criteria	Sciatica; Serious pathology; Unable to attend the exercise classes; Other musculoskeletal activities affecting their ability to cope with the fitness programme; concurrent physiotherapy; major recent surgery; systemic conditions; Spondylolisthesis; engaged in moderately strenuous sporting activities at least twice a week for the previous 6 months; Pregnancy	Possible spinal pathology; Severe or progressive motor weakness or central disc prolapse; Past spinal surgery; Pending litigation; Bleeding disorders; Currently receiving acupuncture treatment.
Financial incentives	None	*£*5 per patient referred, as compensation for time taken explaining the trial to patients
GP trial advisor	No	Yes
GP recruitment	GPs practices were approached by a letter of introduction from the study team, including a copy of the protocol, procedure and background information. Telephone contact was made with the practice manager and a personal visit was arranged by the project leader in order to explain the study in more detail. Emphasis was placed on the need for all GPs plus the practice manager to be present. A total of 19 practices were recruited in this way, with 87 GPs referring patients.	A GP advisor established initial contacts with a number of individual GPs (*n* = 28), and told them to expect a phone call from the study researcher. The researcher telephoned the GPs and arranged a personal visit to explain the study in more detail. The remaining GPs in York (*n* = 98) were sent a letter of introduction from the study team, inviting them to participate in the study. Fifteen GPs returned a postcard indicating their interest and a personal practice visit was arranged. A total of 39 GPs from 18 practices were recruited.
Patient recruitment	One practice provided a computerised list of names of patients who had recently presented with low back pain. Patients were contacted by letter to assess their suitability for the trial and to gain consent. The other 18 practices manually recorded referrals after the consultation by the GP. Over 24 months, a total of 187 patients were recruited out of a target of 300.	Patients were referred directly to the study researcher immediately after a consultation with their GP for low back pain. Over 18 month's period, a total of 289 patients were referred by the GPs, of whom 241 were recruited to the trial.
Strategies for maximising recruitment	Project logo, project updates, referral forms, project reminder letters, personal practice visits.	Project logo, project updates, patients' acknowledgement letters, patient discharge letters, project reminder letters, and personal phone calls to study GPs.

**Table 2:** Practice characteristics of the 8 most successful recruiting practices.

GP practice	Characteristics of practices identified as enhancing recruitment of patients	Exercise trial (Patients recruited)	Acupuncture trial (Patients recruited)	Total recruited (both trials)
A	Special interest in back pain and Computerised referrals to Exercise trial	83	29	111
B	Personally known to researchers	26	49	75
C	Special interest in back pain	—	39	39
	BMAS* member			
D	Acupuncture trial GP advisor	8	28	30
E	Special interest in back pain	—	25	25
F	BMAS* member	9	13	22
G	Special interest in back pain	10	—	10
H	Special interest in back pain	10	—	10

Total	—	**146**	**183**	

*British Medical Acupuncture Society.

**Table 3:** Factors that impact on GPs willingness to refer patients into the trials.

	Exercise trial (*n* = 64)	Acupuncture trial (*n* = 27)
Main factors reducing GPs willingness to refer patients		

Patients only had a 50% or	23	7
66% chance of being offered exercise/acupuncture		
Personal time constraints within the consultation	18	3
Uncertainty of the benefits of exercise/acupuncture	7	1
Patient already receiving other treatment modalities	37	9
Difficulty of patients attending exercise class/acupuncture clinic (travel?)	17	2

Main factors increasing GP willingness to refer		

Desire to support research	49	23
Exercise/acupuncture available as an additional treatment option	—	20
Belief in potential benefits and/or importance of exercise/acupuncture	12	19
Positive feedback from patients who have received treatment	46	18

Feedback on main entry requirements for patients into the study		

Clear (Yes/No)	52/7	27/0
Easy to apply (Yes/No)	43/14	26/1
East to remember (Yes/No)	33/25	22/5

Effectiveness of methods of maintaining a high profile for trial		

Project reminder letters (Yes/No)	49/5	23/3
Project logo (Yes/No)	25/24	20/7
Project updates (Yes/No)	46/9	21/5
Use of the unique referral forms (Yes/No)	48/6	n/a
Personal practice visits (Yes/No)	30/19	n/a
Phone calls from study researcher (Yes/No)	n/a	15/11
Patient acknowledgement letters (Yes/No)	n/a	25/2
Patient discharge letters (Yes/No)	n/a	21/5
Trial information posters in surgery waiting rooms (Yes/No)	n/a	12/12

## References

[1] Rendell JM, Merritt RK, Geddes JR (2007). Incentives and disincentives to participation by clinicians in randomised controlled trials. *Cochrane Database of Systematic Reviews*.

[2] Pearl A, Wright S, Gamble G, Doughty R, Sharpe N (2003). Randomised trials in general practice—a New Zealand experience in recruitment. *New Zealand Medical Journal*.

[3] Ross S, Grant A, Counsell C, Gillespie W, Russell I, Prescott R (1999). Barriers to participation in randomised controlled trials: a systematic review. *Journal of Clinical Epidemiology*.

[4] Hunt CJ, Shepherd LM, Andrews G (2001). Do doctors know best? Comments on a failed trial. *Medical Journal of Australia*.

[5] Lovato LC, Hill K, Hertert S, Hunninghake DB, Probstfield JL (1997). Recruitment for controlled clinical trials: literature summary and annotated bibliography. *Controlled Clinical Trials*.

[6] Pringle M, Churchill R (1995). Randomised controlled trials in general practice: gold standard or fool’s gold?. *British Medical Journal*.

[7] Ward E, King M, Lloyd M, Bower P, Friedli K (1999). Conducting randomized trials in general practice: methodological and practical issues. *British Journal of General Practice*.

[8] Peto V, Coulter A, Bond A (1993). Factors affecting general practitioners’ recruitment of patients into a prospective study. *Family Practice*.

[9] van der Windt DAWM, Koes BW, van Aarst M, Heemskerk MAMB, Bouter LM (2000). Practical aspects of conducting a pragmatic randomised trial in primary care: patient recruitment and outcome assessment. *British Journal of General Practice*.

[10] Borgiel AEM, Dunn EV, Lamont CT (1989). Recruiting family physicians as participants in research. *Family Practice*.

[11] Tognoni G, Alli C, Avanzini F (1991). Randomised clinical trials in general practice: lessons from a failure. *British Medical Journal*.

[12] Murphy E, Spiegal N, Kinmonth A-L (1992). Will you help me with my research? Gaining access to primary care settings and subjects. *British Journal of General Practice*.

[13] Bell-Syer SEM, Klaber Moffett JA (2000). Recruiting patients to randomized trials in primary care: principles and case study. *Family Practice*.

[14] Bryant J, Powell J (2005). Payment to healthcare professionals for patient recruitment to trials: a systematic review. *British Medical Journal*.

[15] Foy R, Parry J, McAvoy B (1998). Clinical trials in primary care: targeted payments for trials might help improve recruitment and quality. *British Medical Journal*.

[16] McCarney R, Fisher P, Van Haselen R (2002). Accruing large numbers of patients in primary care trials by retrospective recruitment methods. *Complementary Therapies in Medicine*.

[17] Watson JM, Torgerson DJ (2006). Increasing recruitment to randomised trials: a review of randomised controlled trials. *BMC Medical Research Methodology*.

[18] Mapstone J, Elbourne D, Roberts I (2007). Strategies to improve recruitment to research studies. *Cochrane Database of Systematic Reviews*.

[19] Klaber Moffett JA, Togerson D, Bell-Syer S (1999). Randomised controlled trial of exercise for low back pain: clinical outcomes, costs, and preferences. *British Medical Journal*.

[20] Thomas KJ, MacPherson H, Thorpe L (2006). Randomised controlled trial of a short course of traditional acupuncture compared with usual care for persistent non-specific low back pain. *British Medical Journal*.

[21] Report of the Clinical Standards Advisory Group on Back Pain.

